# A Sample-Sparing Multiplexed ADCP Assay

**DOI:** 10.3389/fimmu.2019.01851

**Published:** 2019-08-13

**Authors:** Audrey L. Butler, Jonathan K. Fallon, Galit Alter

**Affiliations:** The Ragon Institute of MGH, MIT, and Harvard, Cambridge, MA, United States

**Keywords:** phagocytosis, multiplex, monocyte, antibody-mediated effector profiles, dependent effector function

## Abstract

Antibodies serve as the primary correlate of protection following most clinically approved vaccines and are thought to confer protection in part through their ability to block (neutralize) infection. Increasingly, studies have shown that beyond their blocking activities, the ability of antibodies to leverage the innate immune response may serve a vital role in protection from infection. Specifically, antibodies can drive phagocytosis, complement activation, and cellular cytotoxicity by interacting with Fc-receptors found on all innate immune cells. Measuring the capacity of antibodies to induce these functions has become critical for the identification of correlates of protection in large-scale vaccine trials. Therefore, there is a growing need to develop robust, high throughput assays able to interrogate the functional capacity of innate immune recruiting antibodies. However, in many instances, only small sample volumes are available. Nevertheless, profiling antibody functions across many pathogen-associated antigens or across global intra-pathogen variants is in high demand, making sample sparing approaches to perform this antibody evaluation critical. Here we describe the development of an approach to interrogate the functional activity of antibodies in serum against up to 5 antigen targets simultaneously. A single bead-based cellular assay was adapted to accommodate 5 different fluorescently colored beads, allowing for the concurrent investigation of antibody responses directed against multiple antigens in a single well. The multiplexed assay was as sensitive, specific, and accurate as the single antigen assay and robustly able to assess functional differences mediated by antibodies across different samples. These findings show multiplexing allows for accurate and more efficient analysis of antibody-mediated effector profiles.

## Introduction

Antibodies represent the primary correlate of protection following most clinically approved vaccines (1, 2), particularly for their ability to neutralize pathogens. In addition to their ability to block pathogen entry, recent research into mechanisms of antibody-mediated protection against disease has highlighted the importance of non-neutralizing functions of antibodies as critical components of immunity (3–9). Specifically, several correlates of protection studies in both human and non-human primate HIV vaccine trials have shown the significant relationship between innate immune effector functions and protection (10–15). Similarly, Fc-mediated antibody functions, such as phagocytosis and NK cell activation, have been implicated in protection against Ebola virus (16). Moreover, antibody-dependent cellular cytotoxicity (ADCC) is known to contribute to viral control in influenza infection (17), and protection is largely dependent on Fcγ receptor interactions (18, 19). Non-neutralizing innate immune cell functions are also important for non-viral antigens, as protection against bacterial toxins has been shown to require FcγR engagement (20). These data underscore the emerging importance of functional antibodies in protection across many pathogens and types of infections. They also highlight the need for assays able to interrogate these functions to guide next generation rational vaccine design aimed at harnessing Fc-mediated innate immune cell functions.

Toward this end, techniques to assess functional antibody responses have been critical in the evaluation of vaccine candidates and defining correlates of protection against HIV and other diseases (21–23). Several techniques have been developed to measure ADCC (24–29), antibody-dependent cellular phagocytosis (ADCP) (3, 30, 31), and antibody-dependent complement deposition (ADCD) (32, 33). However, with the growing need to assess antibody functionality across pathogen-variants or different viral and bacterial antigens in large cohorts with limited sample volumes, the need to maximize data output while minimizing reagent input is urgently needed. Techniques allowing for simultaneous interrogation of functional activity of antibodies against multiple antigens could significantly limit the total amount of sample needed to accelerate the identification of functional, antibody-mediated correlates of protection.

Here we optimized a multiplexed approach to assess phagocytic activity across 5 antigens simultaneously. With this new technique, five times the amount of data can be captured using a fraction of the original sample cost with accelerated speed. This assay maintains the specificity, sensitivity, accuracy, and robustness of the non-multiplexed technique, while allowing for faster and more resource-efficient investigation of the potential for vaccine candidates to elicit phagocytic activity in many different disease states. The new approach presented here offers a step forward for improving the study of systems immunology on limited samples and will further propel our understanding of antigen-specific functional humoral correlates of protection from infection across diseases.

## Materials and Methods

### Patient Sample Cohort

A set of 73 HIV-infected serum samples was profiled, including 13 elite controllers [viral loads (VL) ≤ 40 copies of RNA/mL)], 27 viremic controllers (VL 40-2000 copies of RNA/mL), 21 HIV-positive patients on combination antiretroviral therapy (cART, VL ≤ 40 copies of RNA/mL), 12 HIV-positive untreated progressors (VL > 2000 copies of RNA/mL), with 20 seronegative controls. Samples were a subset of a larger cohort (24). All subjects provided informed consent, and the study was approved by the Partners Internal Review Board and by the Partners Human Research Committee.

### Cells, Viral Antigens, Fluorescent Beads, and Monoclonal Antibodies

THP-1 cells were purchased from ATCC and maintained in RPMI 1640 media (ATCC) containing 2 mM L-Glutamine (Corning), 10% Fetal Bovine Serum (Sigma), 10 mM HEPES (Corning), 55 μM beta-mercaptoethanol (Gibco), and 1X Penicillin/Streptomycin (Corning). Cell culture densities were kept below 0.5 × 10^6^ cells/ml to maintain consistent assay performance.

The following purified protein antigens were purchased from Immune Technology Corp.: HIV gp120 (SF162, IT-001-0028p), HIV gp41 (HXBc2, IT-001-005p), HIV p24 (HXBc2, IT-001-017p), influenza hemagglutinin (HA, A/New Caledonia/20/99, IT-003-001p), herpes simplex virus 1 (HSV-1) gD (IT-005-055), and HSV-2 gC (IT-005-011). Recombinant Ebola glycoprotein was purchased from IBT Bioservices (0501-15) and used as a negative control antigen.

Fluorescent, carboxylate-modified 1 μm beads of the following colors were purchased from Thermo Fisher: blue (365/415 nm, F8814), yellow-green (505/515 nm, F8823), crimson (625/645 nm, F8816), red (580/605 nm, F8821), and nile red (535/575 nm, F8819).

Monoclonal antibodies (mAbs) specific for HIV gp120 (2G12), HIV gp41 (2F5), and influenza HA (CH65) were acquired from the NIH AIDS Reagent Program. A monoclonal antibody (c13C6 FR1) recognizing the Ebola virus glycoprotein (GP) was purchased from IBT Bioservices (0201-023).

### Protein Antigen Coupling to Fluorescent Beads

Protein antigens were covalently coupled to fluorescent beads via a two-step carbodiimide reaction. The beads were activated with 80 μL of activation buffer (0.1M NaH_2_PO_4_, pH 6.2), 10 μL of 50 mg/ml Sulfo-NHS (N-hydroxysulfosuccinimide, Pierce, A39269), 10 μL of 50 mg/mL ethyl dimethylaminopropyl carbodiimide hydrochloride (EDC) and incubated for 30 min at room temperature. The beads were washed three times in coupling buffer (0.05 M morpholinoethanesulfonic acid (MES), pH 5.0), then incubated with protein antigen in coupling buffer for 2 h at room temperature. The beads were subsequently washed and blocked with PBS-TBN (PBS, 0.1% BSA, pH 7.4), then washed with PBS-Tween Buffer (PBS, 0.1% BSA, 0.02% Tween 20, 0.05% Azide, pH 7.4). Beads were resuspended in 1 mL of 5% BSA/PBS, incubated overnight at 4°C, and washed and resuspended in 1 mL PBS.

### The Multiplexed Antibody-Dependent Cellular Phagocytosis Assay

A single antigen/single fluorescent bead-based assay to measure ADCP has been described previously (3). For multiplexing experiments, each antigen of interest was coupled separately to fluorescent beads of a distinct color. The antigen-coupled beads were then combined, diluted in 1 mL PBS per assay plate, and added to round bottom 96-well plates so that each well contained 1.8 × 10^6^ beads of each color in a 10 μL volume. A volume of 10 μL diluted human monoclonal antibody or plasma sample was added to each well, and immune complexes were formed over a 2 h incubation at 37°C. After washing to remove non-specific unbound antibody, THP-1 cells were added (200 μL/well) at a concentration of 1.25 × 10^5^ cells/mL (2.5 × 10^4^ cells/well) and incubated with the immune complexed beads for 16 h at 37°C. Cells were then fixed with 4% PFA and acquired on a BD LSRFortessa flow cytometer. Phagocytosis results are analyzed in FlowJo software and reported as a phagocytic score, whereby the geometric mean fluorescence intensity (gMFI) of the bead-positive cells is multiplied by the percentage of bead-positive cells. This value is then divided by 10,000.

### The Multiplexed Antibody-Dependent Neutrophil Phagocytosis Assay

A single antigen/single fluorescent bead-based assay to measure antibody-dependent neutrophil phagocytosis (ADNP) has previously been described (34). For multiplexing experiments, antigens of interest were coupled separately to fluorescent beads of distinct colors. The antigen-coupled beads were then combined, diluted in 1 mL PBS per assay plate, and added to round bottom 96-well plates so that each well contained 1.8 × 10^6^ beads of each color in a 10 μL volume. Diluted human plasma samples were added (10 μL/well) and incubated with the beads for 2 h at 37°C. Primary human leukocytes were isolated from whole blood (collected in anticoagulant citrate dextrose tubes) using ACK red blood cell lysis buffer (150 mM NH_4_Cl, 10 mM KHCO_3_, 0.1 mM Na_2_EDTA), then washed twice in ice cold PBS. The leukocytes were diluted in R10 media to 2.5 × 10^5^ cells/mL, then 200 μL/well was added and incubated with the immune complexed beads for 1 h at 37°C. To specifically measure neutrophil phagocytosis, the cells were then surface-stained with a PE-conjugated CD66b antibody (BioLegend, 305105), fixed with 4% PFA, and acquired on a BD LSRFortessa flow cytometer. Gates were drawn on SSC^high^CD66b^+^ cells, and phagocytic scores were calculated as (% bead-positive cells) x (gMFI (bead-positive cells))/10,000.

### Statistics

Pearson correlations were used to examine bivariate associations. *P* values are two-sided. Comparisons between multiple groups within a cohort were computed by one-way ANOVA adjusted for multiple comparisons using Dunn's test. Statistical analyses were conducted using GraphPad Prism software.

## Results

### The Multiplexed Phagocytosis Assay

**Figure 1:**
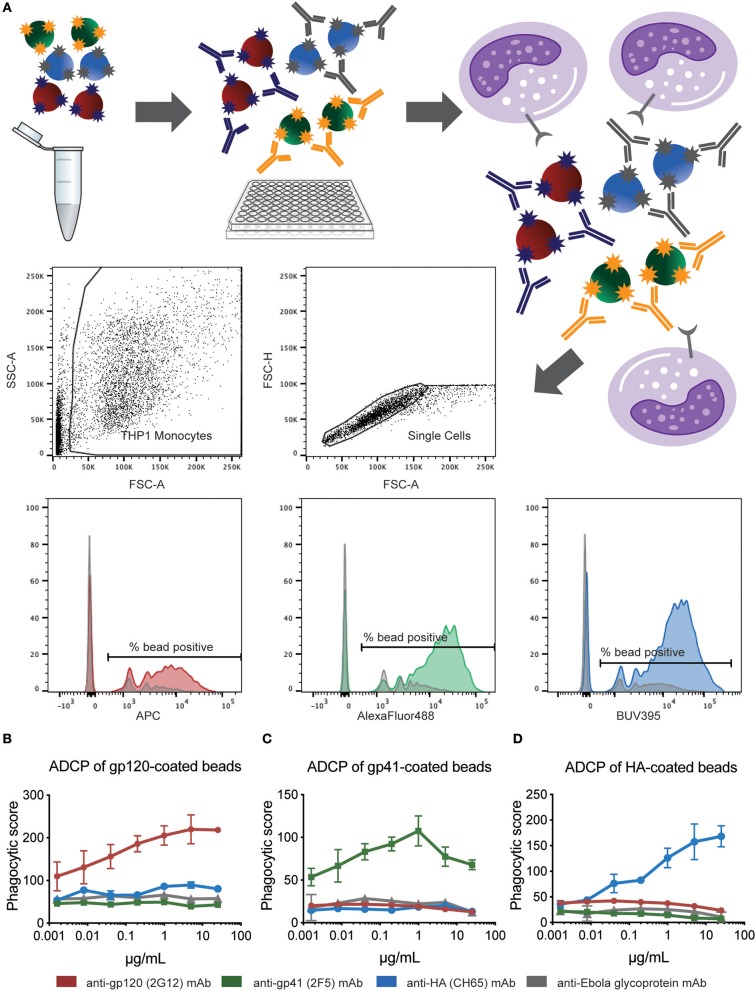
Multiplexed assay setup and antigen-specificity. **(A)** HIV gp120, HIV gp41, and influenza HA proteins were carboxyl-coupled to 1 μm crimson, yellow-green, and blue beads, respectively. These antigen-coupled bead sets were then combined, incubated with 0.0016-25 μg/ml gp120-, gp41-, HA-, or Ebola GP-specific mAb, washed, incubated with human THP-1 monocytes, fixed, and analyzed by flow cytometry. Gates were drawn on singlet THP-1 cells, and phagocytic scores were calculated from data on the APC, AF488, and BUV395 fluorescence channels to quantify gp120-, gp41-, and HA-specific ADCP, respectively. Histograms indicate results with 25 μg/ml positive (bead-coupled antigen-specific) and negative control (Ebola glycoprotein-specific; shown in gray) mAbs. **(B–D)** Graphs show phagocytic scores for **(B)** HIV gp120-, **(C)** HIV gp41-, and **(D)** influenza HA-specific ADCP for the indicated mAb titrations. Points represent the mean ± SD of triplicate wells.

In the multiplexed assay, different colored bead sets are coupled to distinct antigens. The beads are then combined at equal ratios and co-incubated with diluted serum or plasma samples to form immune complexes. Excess antibodies are washed away, and human THP-1 monocytes are added to the immune-complexed beads. After overnight incubation, bead uptake by the THP-1 cells is analyzed by flow cytometry, by gating on monocytes and then identifying bead-positive cells (Figure 1A). Given that different colored beads are included in this assay, antibody-mediated bead uptake for each antigen can be quantified. Thus, the multiplexed assay aims to improve the efficiency, while simultaneously conserving clinical sample volumes, of capturing functional differences across antigen specificities within a single well.

### Confirming Antigen-Specificity in Multiplexed ADCP

Since the original ADCP assay (3) uses an optimized ratio of beads to THP-1 monocytes, we initially sought to determine whether the addition of extra non-specific beads would alter antigen-specific bead uptake. To address this possibility, three fluorescent bead sets were coated with distinct antigens (HIV gp120, HIV gp41, influenza HA), and monoclonal antibodies (mAb) specific to each antigen were added individually or in combination. Combining gp120-, gp41-, and HA-coupled beads in the presence of 2G12 (anti-gp120) mAb resulted in increased uptake of only gp120-coated beads over a wide range of antibody concentrations tested (Figure 1B). Gp41- and HA-coated beads were similarly phagocytosed only in the presence of their respective monoclonal antibodies (Figures 1C,D). Comparing phagocytic scores obtained from titrating each monoclonal with those from no-antibody control wells showed that phagocytosis was both antibody-mediated and antigen-specific. No increase in phagocytosis of gp120-, gp41-, and HA-coated beads was observed in the presence of a negative control Ebola-specific mAb as compared to no-antibody control wells. Thus, no phagocytosis above background was mediated by monoclonal antibodies for antigen-coated beads for which the antibodies were not specific (Figures 1B,D), demonstrating that the combination of additional beads does not alter the antigen-specific nature of the phagocytic assay.

### Confirming the Sensitivity of Multiplexed ADCP

Monoclonal antibody-mediated phagocytosis clearly illustrated that the assay retains specificity upon multiplexing. However, whether the assay could detect antigen-specific bead uptake using polyclonal serum or plasma samples remained uncertain. A potential concern was that samples having high antibody titers specific for some antigens but low titers for others in a multi-bead-set panel might affect the wide linear range of detection for the antigens with low-titer antibodies. Such high-titer antibodies might outcompete low-titer antibodies against another antigen for Fc receptor binding and mask those low-titer antibody-mediated phagocytic effects. To address this possibility, ADCP was assessed against 3 antigen-coupled bead sets using pooled mAbs as a pseudo-polyclonal sample.

**Figure 2:**
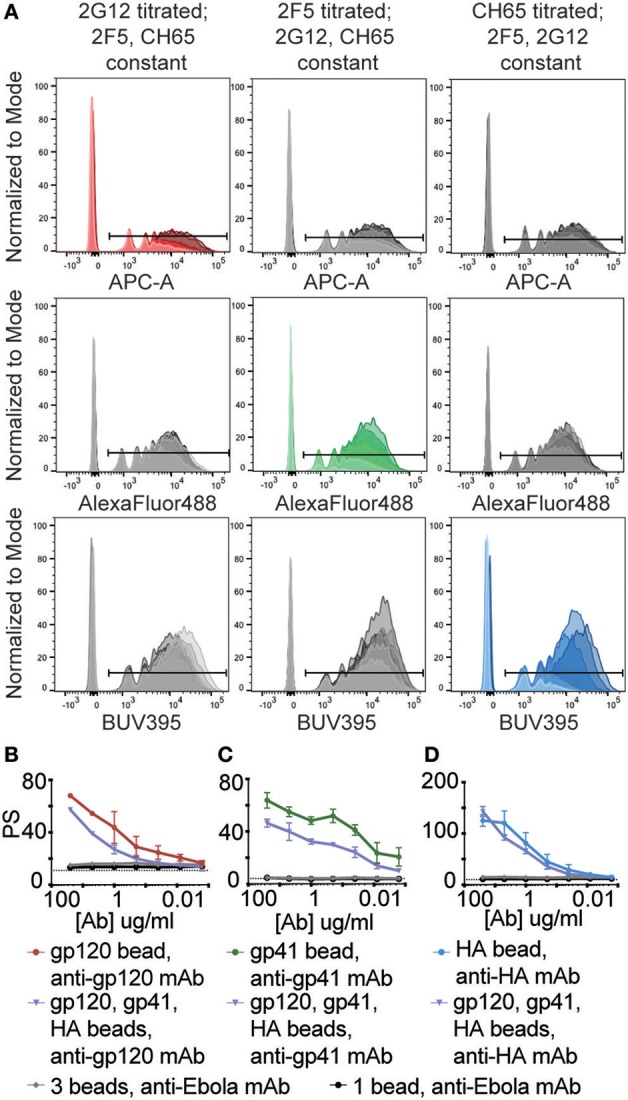
Combining different immune complexed beads does not induce non-specific uptake or alter assay sensitivity. HIV gp120, HIV gp41, and influenza HA proteins were coupled to 1 μm crimson, yellow-green, and blue beads, respectively. These antigen-coupled bead sets were then combined and used in the multiplexed ADCP assay. Antibody samples consisted of gp120-, gp41-, and HA-specific mAb pools in which one mAb was titrated from 0.0016-25 μg/ml and the other two were used at 5 μg/ml. Wells containing 5 μg/ml anti-Ebola GP mAb served as a negative control. **(A)** Histograms show uptake of each fluorescent bead set by THP-1 cells for 3 conditions: (left column) 2G12 mAb titrated with 2F5 and CH65 mAb constant; (middle column) 2F5 titrated with 2G12 and CH65 constant; and (right column) CH65 titrated with 2G12 and 2F5 constant. Red, green, and blue colored histograms indicate that the titrated mAb used is specific for the antigen coupled to that fluorescent bead set, and markers indicate cells that have taken up beads. **(B–D)** Graphs depicting the phagocytic scores (PS) vs. mAb concentration for **(B)** gp120-, **(C)** gp41-, **(D)** HA-specific ADCP for the indicated antigen-coupled bead sets and mAbs. The dotted lines represent background PS levels (wells containing 0 μg/ml of titrated mAb, 5 μg/ml each of the two irrelevant mAbs). Points represent the mean ± SD of triplicate wells.

For multiplexed wells (3 antigen-coupled bead sets and 3 mAbs), one mAb was serially diluted while the other two mAbs were kept at a concentration of 5 μg/ml across the same wells. With respect to assay sensitivity, all antigens demonstrated a step-wise reduction in the percent of bead-positive monocytes as the respective antibody was titrated (Figure 2A). Irrespective of the antibody concentration, ADCP remained antigen-specific and titratable across all 3 antigens (Figures 2B,D). Background was defined here as the level of phagocytosis induced by the negative control Ebola mAb.

While the magnitude of the phagocytic activity varied across single and multiplexed assays, comparable trends within the linear ranges of ADCP were observed for each antibody/antigen pair (Figures 2B,D). However, while the linear range trends for CH65 remained consistent even when gp120- and gp41- specific antibodies were kept at a high concentration (Figure 2C), the levels of phagocytosis for both 2G12 and 2F5 were affected in the multiplexed format (Figures 2B,C), whereby the scores were significantly lower across the different antibody concentrations. Differences in the absolute magnitude of phagocytosis may be related to immune complex mediated competition on THP1 cells that are simultaneously exposed to three different immune complexes in the multiplexed assay. Moreover, despite the fact that all antibodies were produced in 293T cells, each antibody may vary slightly in Fc-glycosylation, thereby resulting in different immune complex affinities that may compete for Fc-receptor binding when immune complexes are mixed in the functional assay. Additionally, orientation of antibody binding, stoichiometry (35), and shape and size of immune complexes (36) may also qualitatively affect Fc-receptor engagement and competition. Thus, future efforts aimed at comparing phagocytic activity across monoclonal antibodies may consider Fc-glycosylation and evaluate immune complex size and quality to account for variation in the single and multiplexed assays. However, immune complex mediated competition within polyclonal sera likely reflects the functional activity within an infected individual, possibly providing a more relevant measure of functionality compared to what may be observed using single bead/antigens alone.

### Confirming Accuracy of 3-5 Bead set ADCP

**Figure 3:**
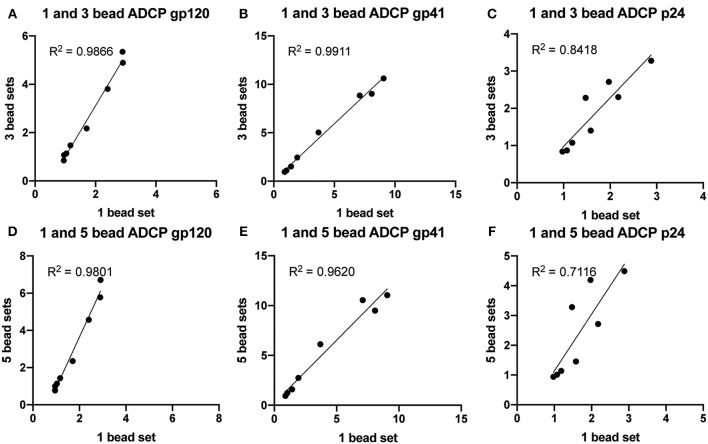
ADCP is highly correlated between multiplexed and single-bead assays. Blue, yellow-green, crimson, red, and nile red 1 μm fluorescent beads were coupled to HIV p24, HIV gp41, HIV gp120, influenza HA, or a mixture of HSV-1 gD and HSV-2 gC antigens, respectively. One HIV-positive serum sample was serially diluted and tested in the ADCP assay using one HIV antigen-coupled bead set alone, all three HIV antigen-coupled bead sets combined, or all five antigen-coupled bead sets combined. Each serum sample dilution was tested in quadruplicate and phagocytic scores were averaged, then normalized by dividing by the PS for an HIV-negative control serum sample. Each point represents the average, normalized PS for a single serum sample dilution (1:100, 1:250, 1:625, 1:1562, 1:3906, 1:9765, 1:24414, or no antibody control). **(A–C)** Normalized phagocytic scores are shown for **(A)** gp120-, **(B)** gp41-, and **(C)** p24-specific ADCP using 1 or 3 HIV antigen-coupled bead sets in a single well. **(D–F)** Normalized phagocytic scores are shown for **(D)** gp120-, **(E)** gp41-, and **(F)** p24-specific ADCP using 1 or 5 antigen-coupled bead sets in a single well. R2 values were computed with linear regression Pearson correlation with a 95% confidence interval.

Next, we aimed to determine whether including additional bead sets could further expand the multiplexed assay. ADCP was therefore tested with 1, 3, and 5 different antigen-coated bead sets. Beads were coupled to HIV gp120, gp41, p24, influenza HA, or a mixture of HSV-1 gD and HSV-2 gC proteins. One HIV-positive plasma sample that also contained high titer influenza and HSV antigen-specific antibodies was serially diluted and used as the source of polyclonal antibodies. ADCP was measured using one set of HIV antigen-coupled beads, three sets of HIV antigen-coupled beads, or all five sets of viral antigen-coupled beads. Across a ~250-fold sample dilution range, there was a strong linear relationship between phagocytic scores in the multiplexed and single-bead assays for gp120 (Figures 3A,D), gp41 (Figures 3B,E), and p24 (Figures 3C,F). This result was consistent for assays run with three (3A-C) or five antigen-coupled bead sets (3D-F). Importantly, variation in correlation strength, ranging from 0.7 to nearly 1, fell within the expected and accepted 30% coefficient of variation range for cell based assay variability. Overall, the multiplexed ADCP assay was specific and accurate compared to the original single-antigen/single-bead protocol.

### Multiplexing With Clinical Samples From HIV Controllers and Progressors

To further probe the accuracy of the phagocytic results captured in the multiplexed ADCP assay compared to the single-bead assay, serum samples from a cohort of 73 HIV-infected patients comprising four HIV clinical phenotypes with 20 HIV seronegative controls were used to test whether multiplexed ADCP could be used to accurately detect differences in antibody function between clinically distinct groups. This cohort included a mix of elite controllers (spontaneous controllers of HIV off antiretroviral therapy (ART) with undetectable viral loads), viremic controllers (spontaneous controllers of HIV off ART with detectable viral loads, >50-2000 copies of RNA/ml), HIV-infected patients treated with ART (<50 copies of RNA/ml), HIV-infected untreated patients (chronic progressors with detectable viral loads, >50 copies of RNA/ml), and HIV-seronegative controls. Because the variation in the degree of viral control between these groups might indicate distinct antibody response levels against HIV antigens, single-bead, and multiplexed ADCP assays were conducted to measure gp120-, gp41-, and p24-specific ADCP activity.

**Figure 4:**
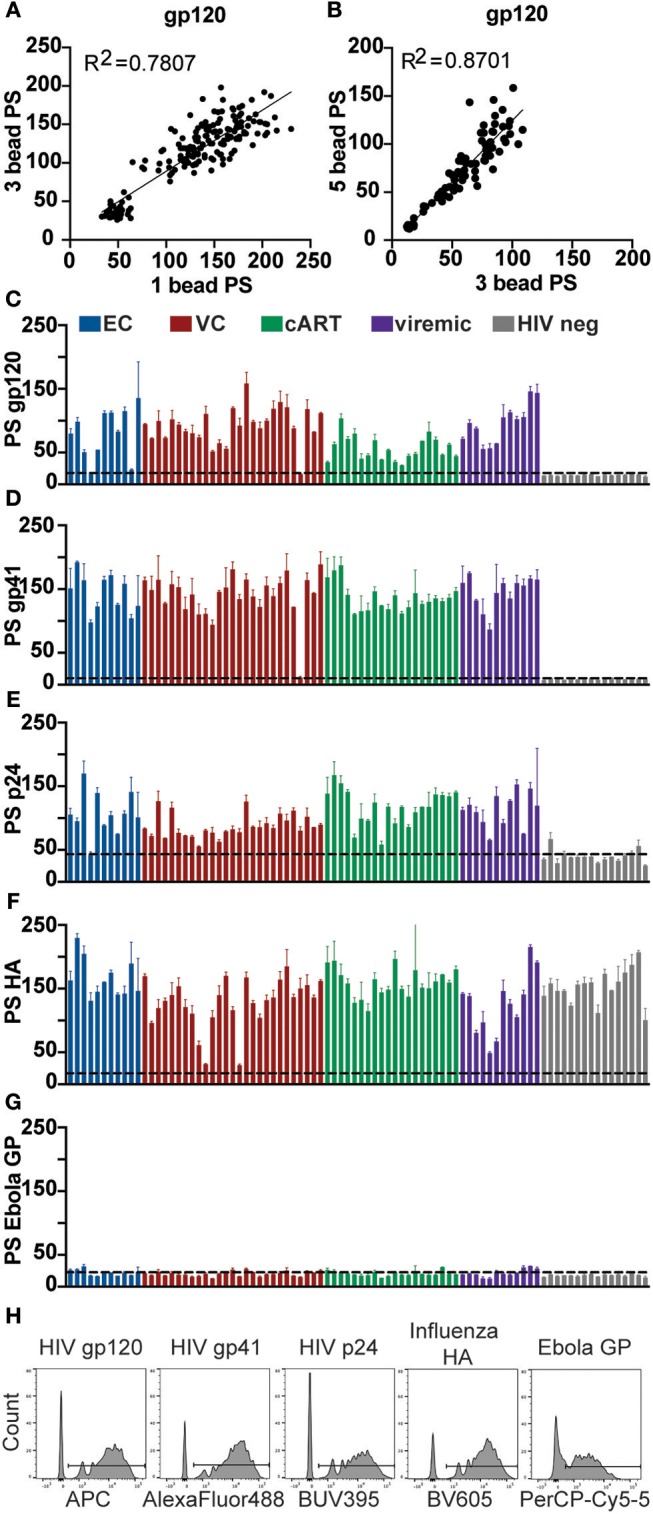
Phagocytosis induced by HIV antibodies in clinical samples is antigen-specific and highly correlated between single and multiplexed bead assays. Crimson, yellow-green, blue, red, and nile red 1 μm fluorescent beads were coupled to HIV gp120, HIV gp41, HIV p24, influenza HA, or Ebola GP antigen, respectively. Each serum sample from a cohort of 73 HIV-positive subjects with 20 HIV seronegative controls was diluted 1:100 and tested in the ADCP assay using the gp120-coupled bead set alone, all three HIV antigen-coupled bead sets combined, or all five antigen-coupled bead sets combined. Each serum sample was tested in duplicate and phagocytic scores were averaged. **(A)** Phagocytic scores are shown for gp120-specific ADCP using 1 vs. 3 HIV antigen-coupled bead sets in a single well. Each point represents one serum sample. **(B)** Phagocytic scores are shown for gp120-specific ADCP using 3 HIV antigen-coupled bead sets vs. all 5 viral antigen-coupled bead sets in a single well. Each point represents one serum sample. R2 values were computed with linear regression Pearson correlation with a 95% confidence interval. **(C–G)** Phagocytic scores (PS) are shown for **(C)** gp120-, **(D)** gp41-, **(E)** p24-, **(F)** influenza HA-, and **(G)** Ebola GP-specific ADCP using all 5 antigen-coupled bead sets in a single well. HIV seronegative cohort samples are grouped at the right end of each graph. Each bar represents the mean ± SD of duplicate wells for one serum test sample. The dashed line indicates phagocytic score for a Massachusetts General Hospital (MGH) HIV-seronegative sample **(C–E)** or no-antibody control **(F,G)**. **(H)** The histograms show the fluorescent bead uptake for each bead set in the assay panel for a representative HIV-positive sample.

HIV gp120, HIV gp41, HIV p24, influenza HA, and Ebola GP antigens were each coupled to a different 1 μm fluorescent bead set. Each serum sample was tested in the ADCP assay using the gp120-coupled bead set alone, all three HIV antigen-coupled bead sets combined, or all five antigen-coupled bead sets combined. Consistent with findings from prior experiments, the phagocytic scores did not differ significantly for individual samples whether gp120, gp41, and p24-coated beads were assayed together in a single well or separately. ADCP results from single and multiplexed 3 bead set assays were significantly correlated (Figure 4A), indicating that the multiplexed ADCP assay improves efficiency without a loss in accuracy. The observed variation in phagocytic scores for individual samples between single and triple bead-set assays remained within the assay variability normally observed between single-bead ADCP assay runs or technical replicates. Additionally, phagocytic scores from the 3 bead set ADCP assay were highly correlated with results from the 5 bead set ADCP assay (Figure 4B) using the same serum samples, further depicting the robustness and accuracy of the multiplexed assay.

Next, the full 5-bead capacity of the assay was used to compare phagocytic activity across additional positive and negative control antigens. HIV antigens gp120, gp41, and p24 were included as antigens for which antibody titer and function was already known to vary across individuals. In addition, influenza HA was used as a positive control against which almost all samples in the cohort should elicit responses. An Ebola glycoprotein (GP) antigen was used as a negative control for which no samples in the cohort should contain cross-reactive antibodies. Phagocytic activity for HIV antigens fluctuated across samples, as expected, and were not detected above assay background levels in the HIV seronegative and no antibody controls (Figures 4C–E). Influenza HA-specific Fc-mediated phagocytic activity was high across most samples (Figure 4F), with a few exceptions, as expected based on variability in immunity in the general population. As expected, no samples elicited responses above the assay background levels against the Ebola antigen (Figure 4G). Bead uptake for a representative HIV-positive serum sample across all five antigens is shown in Figure 4H.

**Figure 5:**
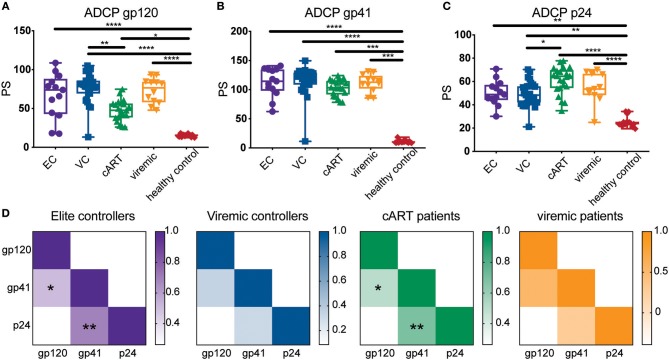
The multiplexed ADCP assay reveals trends in HIV antigen-specific antibody responses in HIV controllers and progressors. Serum samples from a cohort of HIV-infected elite controllers (EC), viremic controllers (VC), cART-treated HIV positive patients (cART), HIV-positive progessors (viremic), and HIV seronegative controls were diluted 1:100 and tested in the ADCP assay using gp120-, gp41-, and p24-coupled fluorescent bead sets. Shown are phagocytic scores for **(A)** gp120-, **(B)** gp41-, and **(C)** p24-specific ADCP for the samples in each clinical subgroup. Each point represents the average PS for one serum sample tested in duplicate. Differences between groups were evaluated using a non-parametric one-way ANOVA adjusted for multiple comparisons using Dunn's test; ^*^p < 0.05; ^**^p < 0.01; ^***^p < 0.001; ^****^p < 0.0001. **(D)** The heat maps depict the median phagocytic scores for each clinical group (ECs, VCs, ART, viremic) across the gp120, gp41, and p24 HIV antigens. Correlation coefficients (r) are represented by color gradients; ^*^p < 0.05; ^**^p < 0.01.

Finally, samples from this experiment were organized into their respective groupings (elite controllers, viremic controllers, cART treated, viremic progressors, and healthy controls), and results from the multiplexed ADCP assay were analyzed for differences in Fc-mediated immune effector function. Antibody function differed significantly between HIV-positive groups and HIV-negative controls across all antigens (Figures 5A–C). Overall, antibody function between most HIV-positive subgroups was similar for all 3 HIV antigens tested, although ART-treated patients showed reduced gp120-specific ADCP (Figure 5A) and increased p24-specific ADCP activity (Figure 5C) compared to viremic controllers. Although the differences between most subgroups were not statistically significant, some variation was found, and these trends were consistent with previously published gp120-specific antibody functionality data using a larger HIV cohort from which the clinical samples here were taken (24), highlighting the ability to capture similar findings in the multiplexed and single bead assays. Moreover, variation was found in the coordination between functional antibody responses among the three HIV antigens, whereby gp41- and p24-specific ADCP responses were significantly correlated for elite controllers and ART patients, as were gp41- and gp120-specific responses (Figure 5D). However, these strong correlations were not significant in viremic controllers or viremic patients, suggesting a less-coordinated overall HIV response in the setting of active viral replication. Overall, the multiplexed ADCP technique expands not only the amount of data collected but also the analyses possible within just one assay.

### Multiplexing Can Be Expanded to Primary Neutrophils

**Figure 6:**
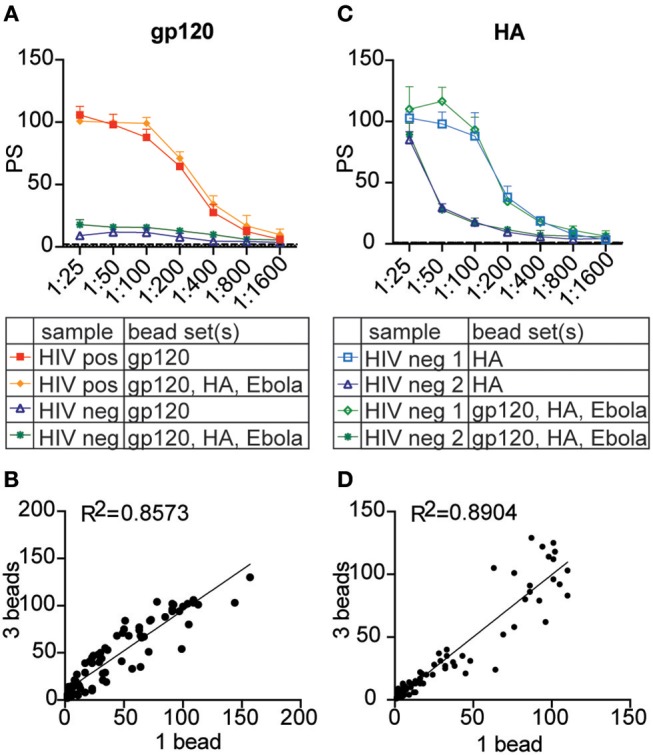
The multiplexed assay can be expanded to primary neutrophils. Crimson, blue, and yellow-green 1 μm fluorescent beads were coupled to HIV gp120, influenza HA, and Ebola glycoprotein antigen, respectively. Three plasma samples from HIV seronegative and positive donors were titrated from 1:25 to 1:1,600 and tested in the phagocytosis assay using each antigen-coupled bead set individually and in the 3-bead multiplexed format. Instead of THP-1 cells, primary leukocytes were used as a source of neutrophils to measure antibody-dependent phagocytosis. **(A,C)** Titration curves show phagocytic scores for gp120- **(A)** and HA- **(C)** specific ADNP in the multiplexed and single-bead formats for representative HIV-positive and HIV-negative plasma samples. Each point represents the mean ± SD of triplicate wells. The dashed line indicates the phagocytic score for a no-antibody control. **(B,D)** Dot plots show phagocytic scores for gp120- **(B)** and HA- **(D)** specific ADNP using 1 vs. 3 bead-sets in a single well. *R*^2^ values were computed with linear regression Pearson correlation with a 95% confidence interval.

To test whether the multiplexed assay could be applied to another cell type, primary neutrophils from donor leukocytes were used in the multiplexed format based on an optimized protocol for testing ADNP (34). Here, HIV gp120, influenza HA, and Ebola GP antigens were each coupled to a different 1 μm fluorescent bead set and incubated with HIV positive and/or negative plasma either alone or with all bead sets combined. Leukocytes were isolated from whole blood by lysing the erythrocytes, and neutrophils were identified by staining with a CD66b-specific fluorescent antibody. As with the monocyte multiplexed assay, antibody-dependent phagocytosis by neutrophils was assessed using a phagocytic score. From this, significant variation in ADNP activity across the antigens was observed and the results showed multiplexing can be expanded beyond the monocyte cell line used to develop and optimize the technique presented here. Phagocytosis scores for gp120- and HA-specific antibodies were comparable between single- and three-bead set ADNP and were strongly correlated (Figure 6). Thus, the multiplexing technique to measure antibody-dependent phagocytosis can be applied not only to a broad range of sample types and antigens, but also to various phagocytic cell types.

## Discussion

There is an emerging appreciation for the critical role for antibody Fc-mediated effector functions in protection across a wide array of infections (37–39). Thus, assays able to systematically capture the innate immune recruiting function of pathogen-specific antibodies are urgently needed. High-throughput, sensitive, and specific assays that can capture information across multiple antigens simultaneously would provide significant advantage with respect to cost and sample volume needs. Here we describe efforts to multiplex a bead-based phagocytic assay aimed at measuring monocyte-mediated, antibody-driven phagocytic activity across 5 antigens simultaneously. Multiplexing did not alter the sensitivity or specificity of the technique, providing an accelerated method to capture 5 times more functional data in a single assay. This sample-sparing approach offers a high throughput manner to investigate monocyte phagocytosis. However, the bead-based approach can be adapted to probe the additional role of other innate phagocytic cells, including primary monocytes, macrophages, dendritic cells, and neutrophils, and can even be used to investigate complement deposition, offering a remarkably broad platform to capture a large amount of data concurrently via the adapted use of multiple fluorescent beads simultaneously.

For many clinical trials, only small amounts of sample can be collected. For example, the collection of blood from neonates (40), mucosal samples (41), cerebrospinal fluid (42), as well as samples from small animal models (43) may limit the number of analyses that may be performed within a given study. Thus, sample-sparing approaches to capture a larger amount of data may profoundly improve our ability to fully dissect the humoral immune response following various types of infection or vaccination. Importantly, while this assay does not reduce the overall volume of serum or plasma required (~1 μl), which is already quite small, the technique provides a means to capture 5 times more data simultaneously from the same sample, providing an opportunity to probe for functional profiles across different antigens. Moreover, multiplexed methods can be used to probe for epitope-specific responses using different epitope-scaffolds (44), linear peptides (45), or even to look for breadth of function across variants of a given antigen (46, 47).

Beyond profiling responses across pathogens and antigens of interest, the assay platform may be expanded to probe epitope specificity and breadth of functional humoral immunity. Specifically, in the context of HIV, influenza, dengue infections, etc., there is great interest in defining the nature of protective humoral immune responses able to recognize global variants (48, 49). Often, conserved epitope specificities at the receptor binding site (50) or in conserved regions of the viral envelopes (51, 52) can be recognized by antibodies, providing protection from infection in a neutralization independent manner (53, 54). In this case, the multiplexing technique could be useful to test panels of envelope isolates from globally relevant and distant strains, extending beyond current ELISA and Luminex simple binding assays to determine the functional properties of antibodies able to bind to multiple antigens. Given that multiple bead colors can be interrogated simultaneously, the uptake of mono- or poly-chromatic bead combinations may provide an unprecedented depth of information on cross-reactive functional responses. Additionally, modified antigens, lacking or mutated in the receptor binding domain (55, 56) or only displaying minimal target epitopes of interest (55, 57) can also be attached to beads to specifically probe the functional properties across the surface of target antigens of interest. Collectively these applications of the multiplexed assay may be linked to inhibitory, enhancing, or microscopic assays to dissect the consequences of bead up take by type specific or cross-reactive antibodies across infections.

The assay presented here exploits 5 bead colors, however up to 7 colors are currently available that can be detected on more sophisticated flow cytometers. Therefore, as additional bead colors are in development, the number of antigens that may be investigated simultaneously will likely increase, offering even greater numbers of specificities that may be concurrently probed. Additionally, instead of single-color fluorescent beads, Luminex beads enable the generation of hundreds of bead sets by exploiting two fluorochrome ratios, extending the number of non-overlapping antigens that can be tested simultaneously on a flow cytometer. Thus, for diseases where the functional antibody antigen/epitope-specificity remains unknown, these additional bead platforms offer an even broader opportunity to capture a large amount of data.

Additionally, the multiplexed phagocytosis assay may be used to investigate pathogen cross-reactivity. For example, it is known the antigenic overlap between different flaviviruses leads to antibody-dependent enhancement of Dengue viral infection through pre-existing non-neutralizing antibodies induced by cross-reactive dengue responses (58). Therefore, the unique nature of the multiplexed technique to expose phagocytic cells to different antigens in a single well-provides an opportunity to explore how dengue, and other flavivirus-specific antibodies, either possibly facilitate, or inhibit uptake of pathogen-specific immune complexes. Moreover, whether immune complex uptake leads to pathogen elimination or facilitation of infection can also be further investigated in these assays, with follow-up analytics. For example, changes in bead localization can be captured by microscopy, antigen presentation may be measured by T cell co-culture, cell maturation/activation may be assessed by flow-cytometry through intracellular cytokine staining (59), and secondary activating signals (cytokines/chemokines) can be quantified by Luminex bead array or ELISA. Thus, additional data may be collected from this multiplexed bead-based approach.

Sample-sparing assays able to reproducibly, specifically, and sensitively capture the functional capacity of antigen-specific antibodies provide a path for the identification of novel correlates of immunity for pathogens where correlates remain to be defined (9, 60), an opportunity to define epitope-specific functional targets of protective humoral immune responses (48), and/or enable the dissection of cross-reactive humoral immune responses (61–63). Together, these insights may have a profound impact on next generation vaccine or monoclonal therapeutic design, guiding the rational design of both the Fab- and Fc-mediated activities of a humoral immune response that may provide the highest level of protective immunity. However, this approach may be exploited to probe humoral immune activity across a much broader array of diseases, and potentially also used to profile and define novel functional humoral specificities in autoimmune, allergic, and even oncological diseases, offering a remarkably flexible platform tool for the dissection of humoral immune responses.

## Data Availability

The raw data supporting the conclusions of this manuscript will be made available by the authors, without undue reservation, to any qualified researcher.

## Author Contributions

AB, JF, and GA were responsible for conception of the research idea, were responsible for planning the study and, were responsible for analyzing the data and writing the manuscript. AB performed experiments.

### Conflict of Interest Statement

The authors declare that the research was conducted in the absence of any commercial or financial relationships that could be construed as a potential conflict of interest.
